# Elevated plasma neurofilament light was associated with multi-modal neuroimaging features in Alzheimer’s disease signature regions and predicted future tau deposition

**DOI:** 10.1186/s12883-024-03728-7

**Published:** 2024-07-06

**Authors:** Qili Hu, Mengqiu Shi, Yunfei Li, Xiaohu Zhao

**Affiliations:** Present Address: Department of Imaging, The Fifth People’s Hospital of Shanghai, Fudan University, No.128 Ruili Road, Minhang District, Shanghai, 200240 China

**Keywords:** Neurofilament light, Multi-modal neuroimaging biomarkers, Tau deposition, Alzheimer’s disease

## Abstract

**Background:**

Neurofilament Light (NfL) is a biomarker for early neurodegeneration in Alzheimer’s disease (AD). This study aims to examine the association between plasma NfL and multi-modal neuroimaging features across the AD spectrum and whether NfL predicts future tau deposition.

**Methods:**

The present study recruited 517 participants comprising Aβ negative cognitively normal (CN-) participants (n **=** 135), Aβ positive cognitively normal (CN +) participants (*n* = 64), individuals with amnestic mild cognitive impairment (aMCI) (*n* = 212), and those diagnosed with AD dementia (*n* = 106). All the participants underwent multi-modal neuroimaging examinations. Cross-sectional and longitudinal associations between plasma NfL and multi-modal neuro-imaging features were evaluated using partial correlation analysis and linear mixed effects models. We also used linear regression analysis to investigate the association of baseline plasma NfL with future PET tau load. Mediation analysis was used to explore whether the effect of NfL on cognition was mediated by these imaging biomarkers.

**Results:**

The results showed that baseline NfL levels and the rate of change were associated with Aβ deposition, brain atrophy, brain connectome, glucose metabolism, and brain perfusion in AD signature regions (*P*<0.05). In both Aβ positive CN and MCI participants, baseline NfL showed a significant predictive value of elevating tau burden in the left medial orbitofrontal cortex and para-hippocampus (β = 0.336, *P* = 0.032; β = 0.313, *P* = 0.047). Lastly, the multi-modal neuroimaging features mediated the association between plasma NfL and cognitive performance.

**Conclusions:**

The study supports the association between plasma NfL and multi-modal neuroimaging features in AD-vulnerable regions and its predictive value for future tau deposition.

**Supplementary Information:**

The online version contains supplementary material available at 10.1186/s12883-024-03728-7.

## Background

### Understanding Alzheimer’s disease (AD) and the need for biomarkers

AD is a prevalent neurodegenerative disorder and a leading cause of dementia [1]. AD is characterized by a gradual decline in cognitive function, particularly episodic memory, and it has been projected that the number of individuals aged 65 and above with AD will reach 13.8 million by 2050. As such, there is a pressing need for early diagnosis of preclinical AD to facilitate timely intervention [2].

The current modalities utilized for tracking the progression of AD are principally reliant on imaging techniques – specifically, volumetric magnetic resonance imaging (MRI) [3] and positron emission tomography (PET) [4] – that facilitate the visual assessment of metabolically active or aggregated Aβ and tau within the brain, as well as cerebrospinal fluid (CSF) biomarkers indicative of Aβ42 and phosphorylated tau [5, 6]. While these imaging biomarkers are valuable, they suffer from limitations in their cost and accessibility, and CSF biomarkers necessitate invasive lumbar puncture. Thus, there is an urgent need for alternative, noninvasive, and cost-effective biomarkers capable of monitoring AD progression in a clinical context, as well as expediting the development of new therapeutic interventions.

### The promise of neurofilament light chain (NfL) as a biomarker for AD

NfL is a promising candidate biomarker for monitoring neurodegenerative processes in AD [7, 8]. NfL is a component of the axonal cytoskeleton and a putative marker of large-caliber axonal degeneration, which is a critical pathological change in neurodegenerative diseases [9, 10]. In multiple sclerosis, variations in NfL are indicative of disease activity, with heightened levels reflecting more active disease phases. Within frontotemporal dementia [11] and amyotrophic lateral sclerosis contexts, an association between escalated NfL levels and disease advancement proposes its utility as a prognostic instrument. Additionally, for Parkinson’s disease (PD), serum NfL levels have been linked with the evolution of clinical PD, uncovering its potential application in the effective management of parkinsonian syndromes.

In the domain of AD specifically, NfL level and its rate of change in plasma are elevated in both sporadic and familial AD and are closely correlated with clinical symptoms and progression [8, 12]. Increased NfL levels have also been linked with various imaging biomarkers, including brain atrophy (hippocampal volume, entorhinal cortical thickness, ventricular volume, and temporal cortical thickness), decreased brain metabolism, and cross-sectional Aβ deposition [7, 13–15].

However, few studies have explored the correlation between NfL and brain connectivity and perfusion, key features of AD. Furthermore, most previous studies have only focused on one or two imaging modalities. An in-depth and systematic examination of the association between plasma NfL and multi-modal neuroimaging biomarkers is still lacking. Additionally, little is known about whether plasma NfL can track AD pathology accumulation in non-demented individuals at high risk for AD.

### Aims of the current study

To address these gaps in the literature, the present study aimed to investigate the potential associations between plasma NfL levels and various multi-modal imaging features, including Aβ pathology, brain atrophy, structural and functional brain connectivity, glucose metabolism, and brain perfusion. Additionally, this study evaluated the predictive ability of baseline NfL concentrations regarding future tau deposition and tested whether the effect of NfL on cognition was mediated by these imaging biomarkers.

Overall, this study seeks to provide a more comprehensive and systematic examination of the relationship between plasma NfL, multi-modal neuroimaging biomarkers, and cognition across the AD spectrum. By doing so, this study may contribute to a better understanding of NfL as a novel biomarker and facilitate its proper use in AD research and therapeutic trials.

## Methods

### ADNI database

The present article utilizes data acquired from the North American Alzheimer’s Disease Neuroimaging Initiative (ADNI) database (adni.loni.usc.edu). ADNI was established in 2004 by a collaboration between the National Institute on Aging, the Food and Drug Administration, private pharmaceutical companies, and not-for-profit organizations with the aim of creating a pioneering public-private partnership. The primary objective of ADNI has been to evaluate whether the amalgamation of serial MRI, PET, other biological indicators, and clinical and neuropsychological evaluations could be used to gauge the development of MCI and early AD. The lead investigator of this enterprise is Michael W. Weiner, MD, VA Medical Center, and University of California, San Francisco. ADNI is the outcome of the concerted efforts of numerous co-investigators from an extensive range of academic institutions and private businesses, with subjects recruited from over 50 locations across the United States of America and Canada. The initial goal of ADNI was to enroll 800 subjects, yet ADNI GO, ADNI2, and ADNI3 have continued the initiative. To date, these three protocols have enrolled over 1500 adults. For additional information, please visit www.adni-info.org.

### Participants

In order to investigate the role of NfL across the AD spectrum, a cohort of subjects consisting of cognitively normal (CN) controls, amnestic mild cognitive impairment (aMCI), and AD patients with baseline plasma NfL data were included in this study. The inclusion and exclusion criteria have been described in detail on www.adni-info.org. Briefly, the participants were enrolled in ADNI2 and fulfilled the following criteria: aged between 55 and 90 years, completed at least 6 years of education, fluent in Spanish or English, and absence of significant neurological disease other than AD. Controls were defined as having Mini-Mental State Examination (MMSE) scores greater than or equal to 24 and Clinical Dementia Rating scale (CDR) score of 0. Participants with aMCI had MMSE score greater than or equal to 24, objective memory loss as evidenced by scores on delayed recall on the Wechsler Memory Scale Logical Memory II, CDR 0.5, preserved activities of daily living, and absence of dementia.

The primary reason for utilizing ADNI2 participants lies in the unique combination of assessments that this phase offered, which were indispensable for our investigation. Specifically, ADNI2 was the only phase where participants underwent a comprehensive suite of NfL measurements in conjunction with a multi-modal neuroimaging including Aβ-PET, 18-FluoroDeoxyGlucose PET (18 F-FDG-PET), Diffusion tensor imaging (DTI), rest-state functional MRI (rs-fMRI), and Arterial Spin Labeled (ASL) approach at that time. Unlike ADNI1 and ADNI GO, which did not include functional MRI scans, and ADNI3, which at the time of our study had not incorporated NfL assessments, ADNI2 presented the necessary data for our objectives.

The aggregation of Aβ is a hallmark pathological feature of AD, and Aβ deposits can occur in individuals who are still cognitively normal [16, 17]. Current research has referred to Aβ positive subjects as the preclinical phase of AD [18]. Therefore, we further stratified CN subjects into Aβ positive CN (CN+) and Aβ negative CN (CN-). Additionally, studies have shown that Aβ biomarker-positive aMCI patients are more likely to have AD pathology and considered to be in the prodromal stage of AD compared to Aβ biomarker-negative aMCI patients [19]. Therefore, only Aβ positive aMCI and AD patients were included in this study. The status of Aβ was evaluated by both cerebrospinal fluid (CSF) and PET. An abnormal PET status was defined as >0.79 standardized uptake value ratio (SUVR) using the composite reference region [20, 21]. A cut-off for CSF Aβ42 was defined as CSF Aβ42<192 ng/L [22–24]. Participants without baseline CSF Aβ42 and Aβ PET data were excluded from the study.

### Measurement of plasma NfL and CSF Aβ42

The collection, processing, and storage procedures for both plasma NfL and CSF Aβ42 have been previously described on the www.adni-info.org website. The plasma NfL concentration was quantified utilizing the ultrasensitive Single-Molecule-Array technology platform developed by Professors Henrik Zetterberg and Kaj Blennow at the University of Gothenburg, Sweden. A combination of monoclonal antibodies and purified bovine NfL as the calibrator was used with all samples measured in duplicate. All measurements were performed by board-certified laboratory technicians who remained blinded to clinical data, using a single batch of reagents. All plasma NfL samples detected were above the limit of detection, and the analytical sensitivity was less than 1pg/ml [25]. The CSF concentrations of Aβ42 were measured in aliquoted samples utilizing an electrochemiluminescence immunoassay on an Elecsys Cobas-e-601 analyzer (Roche Diagnostics, Penzberg, Germany).

### Neuroimaging acquisition and analysis

Detailed information describing imaging data acquisition and processing is available online at www.loni.usc.edu.

#### 18 F-florbetapir (AV-45) PET

The present study employed AV-45 PET to quantify Aβ deposition through the collection of 4 × 5-minute frames from 50 to 70 min after the injection of approximately 15 mCi of tracer. All scans underwent quality control checks, including assessing counts, field-of-view, and subject movement. Subsequently, the standardized SUVR images were created by applying a series of processing steps, which included realigning and averaging the 50–70 min post-injection frames, processing the images to a standard orientation and voxel size, smoothing to a common resolution of 8 mm FWHM, and normalizing the intensity. To achieve normalization, the global cortical mean SUVR, as well as the regional cortical and subcortical SUVR, were calculated using two different normalization methods [26, 27]. Specifically, the global cortical mean SUVR was calculated relative to a composite reference region consisting of the whole cerebellum, brainstem/pons, and subcortical white matter [21], whereas the regional cortical and subcortical SUVR were intensity normalized to the cerebellum. Finally, regional SUVR was extracted for the standardized SUVR images using regions of interest (ROI) derived from the FreeSurfer software packages [28].

#### Structural MRI

The present study utilized conventional structural brain MRI scans obtained from 3-T imaging systems, employing T1-weighted images with a sagittal, volumetric magnetization-prepared rapid acquisition with gradient echo sequence. Before analysis, T1 preprocessing steps were carried out following the ADNI protocol, including correction for distortions due to gradient nonlinearity (Grad warp), intensity non-uniformity (B1), and bias field correction (N3). Cortical and subcortical volumes were quantified using FreeSurfer, version 5.1, by the 2010 Desikan-Killany atlas and 2009 Destrieux atlas [29, 30]. A thorough visual quality control (QC) was conducted and only images with a good overall segmentation in all 9 regions including Frontal, Temporal, Insula, Parietal, Occipital, Cerebral WM, Basal Ganglia, and Hippocampus were included. Additional information regarding the visual QC process can be found in the supplemental materials provided.

Furthermore, the hippocampus is a region considered paramount in supporting episodic memory function and is extensively affected in Alzheimer’s disease pathology. The hippocampus is a complex and heterogeneous region composed of various functionally and anatomically interconnected, yet distinct subfields. Several histopathological studies have suggested differential AD-associated pathological changes among hippocampal subfields. In order to more accurately examine the relationship between plasma NfL and hippocampal abnormality, the hippocampus was subdivided into multiple ROI using the automated hippocampal subfield segmentation tool provided in FreeSurfer, version 5.1. These regions included the hippocampal fissure, subiculum and presubiculum, Cornu Ammonis (CA)1, CA2-3, CA4-dentate gyrus (DG) [31]. Supplementary Fig. 1 illustrates a sample image from a subject.

#### 18 F-FDG-PET

The measurement of glucose metabolism was conducted using 18 F-FDG-PET imaging. The FDG scans were collected on the same day as the AV-45 PET scans, consisting of 6 × 5-minute frames acquired from 30 to 60 min after injection of approximately 5mCi of tracer, and 120 min after injection of PIB. Subsequently, the acquired frames were realigned, averaged, reoriented, resliced to a common grid, and smoothed to a uniform resolution of 8 mm. Pre-processed images were non-linearly registered to an FDG PET template in MNI (Montreal Neurological Institute) space, utilizing the “Old Normalize” tool in SPM12. Spatially normalized images were then utilized to derive SUVR maps through voxel-wise scaling to the average signal in a pons ROI [32].

To identify the most frequently observed pathological hypometabolic regions in aMCI and AD, a set of ROIs were generated based on regions commonly reported in the literature to exhibit differences between patients with AD and controls. These regions included the bilateral angular gyrus, posterior cingulate/precuneus, and inferior temporal cortex and were defined utilizing coordinates from the Montreal Neurological Institute atlas, and merged into a single composite region [33]. The average SUVR was extracted from the composite region for further analysis.

#### ASL

MRI was performed on 3.0 Tesla MR scanners from a single vendor (MAGNETOM Trio, Verio, Skyra, Siemens). Pulsed ASL scans were collected using QUIPS II with thin-slice we as follows: inversion time of arterial spins (TI1) = 700 ms, the total transit time of spins (TI2) = 1,900 ms, tag thickness = 100 mm, tag to proximal slice gap = 25.4 mm, repetition time = 3,400 ms, echo time = 12 ms, the field of view = 256 mm, matrix = 64 × 64, slice number: 24 (axial), thickness = 4 mm thick axial slices [52 tag + control image pairs], time lag between slices = 22.5 ms.

ASL data processing involved automated motion correction, aligning each ASL frame to the first frame using a rigid body transformation, and least squares fitting using SPM 8 as described previously. The difference between the mean-tagged and mean-untagged ASL images was for the perfusion-weighted images. The images were intensity scaled to account for signal decay during acquisition and to generate intensities in meaningful physiological units. ASL images were aligned to structural T1 images using FSL after geometric distortion correction. A partial volume correction was performed that assumed that CBF in gray matter is 2.5 times greater than in white matter to mitigate the effects of lower perfusion in white matter on cerebral blood flow (CBF) estimates. These images were normalized by the reference image (i.e., an estimate of blood water magnetization) to convert the signal into physical units (mL/100 g tissue/min). After, a global pass/fail rating was given based on visual inspection of signal uniformity, geometrical distortions, gray matter contrast, and the presence of large artifacts for ADNI quality control purposes. A rating of “unusable” in any of these categories resulted in a global “fail” and that participant was excluded from this study. To extract regional CBF estimates for each participant, FreeSurfer-derived anatomical ROIs were applied to CBF maps.

#### DTI

DTI is a powerful tool for investigating the microstructural properties of white matter tracts. By applying measures such as fraction anisotropy (FA), mean diffusivity (MD), axial diffusion (AD), and radial diffusion (RD), it is possible to assess white matter integrity and myelination. In the present study, we utilized the JHU DTI template [34] to register each subject, with the exception of 4 ROIs that were excluded due to partial or complete out-of-field view. In addition to the 52 JHU labels, we evaluated 5 additional ROIs, including the bilateral fornix, bilateral genu, bilateral body, and bilateral splenium of the corpus callosum, as well as the full corpus callosum, to obtain comprehensive summary measures of these regions. All ROIs were subsequently registered to the segmented atlas. Visual inspection of the images was performed to ensure adequate registration. The mean voxel value for each ROI for the maps of FA, MD, AD, and RD were obtained to analyze the data.

#### Rs-fMRI

##### Data acquisition

All subjects were examined using a 3.0-Tesla MRI scanner, manufactured by Philips medical system. One T1-weighted image was acquired for each subject, using a pulse sequence (SPGR) with the following parameters: TR = 3000ms, TE = 30ms, matrix size = 64.0 × 64.0, slice thickness = 3.3 mm, yielding up to 6720 slices and other valid slices.

##### Data preprocessing

MRI data analysis was carried out using Data Processing Assistant for Resting-State fMRI Advanced Edition (DPARSFA V5.3) (http://rfmri.org/DPARSF), based on MATLAB R2013b platform. The initial 10 volumes were discarded to eliminate the subjects’ unstable and volatile magnetic field at the beginning of the scan. All data were then realigned to correct the head motion. Reorienting functional and T1 images to increase the accuracy of co-registration, segmentation, and normalization. Besides segmentation, nuisance covariates regression with white matter and cerebrospinal fluid, Friston 24 head motion parameters as regressors. The functional images were normalized by using T1 image unified segmentation. The normalized images were checked by visual inspection, and 2 subjects were excluded due to poor registration and normalization. The data were spatially smoothed (Gaussian kernel of 6 mm full width at half maximum). Then the time series were filtered with a band-pass filter (0.01–0.08 Hz). After preprocessing, 1 individual’s head motion above 3 mm was removed from the research.

##### ROI-based functional connectivity (FC) analysis

In our study, we utilized two atlases to analyze cortical and subcortical regions, namely the Destrieux atlas with 74 region parcellations [30] and the Choi atlas with 8 region parcellations, respectively. Both atlases were aligned to the Montreal Neurological Institute 5 (MNI) space and merged to create an 82-region atlas. These 82 regions were used as ROIs to extract the BOLD signal time courses. FC was then calculated by analyzing the temporal correlation of rs-fMRI BOLD signal time courses across the 82 ROIs for each participant. There are different many candidates to calculate FC, such as the tangent method and partial correlation; however, we used Pearson’s correlation coefficient because it is the most commonly used in previous studies. We calculated Fisher’s z-transformed Pearson’s correlation coefficients between the preprocessed BOLD signals of each possible pair of ROIs and used them to construct 82 × 82 symmetrical connectivity matrices in which each element represents a connection strength between 2 ROIs. In total, 3362 FC values [(82 × 82)/2] of the lower triangular matrix of the connectivity matrix were used for further analysis.

#### Flortaucipir (AV-1451) PET

The current study utilized AV-1451 PET for the assessment of tau pathology. The image analysis protocol involved the acquisition of one or more Flortaucipir scans, coupled with one or more structural MRI scans, for each participant. The MRI scan that was closest in temporal proximity to each PET scan was subjected to segmentation utilizing Freesurfer software (version 7.1.1) to delineate ROI in the individual’s native space. Subsequently, Flortaucipir scans were co-registered to their corresponding bias-corrected T1 images generated by Freesurfer, and the mean uptake of Flortaucipir in each region was computed by using the inferior cerebellar gray matter as a reference region.

### Clinical and cognitive assessments

Among the clinical tests obtained from ADNI participants, the Alzheimer’s Disease Assessment Scale Cognition 13-item scale (ADAS13) was selected for its comprehensive evaluation of global cognitive function and its established use in clinical trials of AD. This instrument assesses core cognitive domains such as language, memory, praxis, and comprehension, which are relevant to AD, and are constructed from written and verbal responses. The composite score of 13 items is reported on a scale of 0 to 85, with higher scores indicating poorer cognitive function [35]. To mitigate practice effects, different forms of the test were administered at each visit.

### Statistical analyses

#### Demographic information comparison

In this study, statistical analyses were conducted to evaluate various aspects of the data. Initially, baseline demographic and clinical characteristics across groups were compared using either ANOVA and subsequent post hoc tests or the Kruskal-Wallis test where data distribution was not normal or failed to meet the variance homogeneity criteria.

#### Plasma NfL concentration across the AD spectrum

Differences in baseline plasma NfL concentrations among groups were assessed using analysis of covariance. Within the validation set comprising both CN + and CN- groups, baseline plasma NfL concentrations were compared using a paired sample T-test. Furthermore, the NfL cutoff for identifying neurodegenerative disorders across all ages was determined to be 35.02 pg/mL (90% CI), as established in a multicenter validation study evaluating the diagnostic value of plasma NfL [36]. The concentrations in each group were compared with this normal cutoff using a one-sample t-test for validation.

#### Cross-sectional association between NfL and multimodal neuroimaging markers

To accurately gauge the association between plasma NfL levels and multimodal neuroimaging markers, we conducted both cross-sectional and longitudinal analyses. For cross-sectional analyses, partial correlation analysis was used to explore correlations between plasma NfL levels and amyloid-beta (Aβ) deposition, FDG SUVR, DTI metrics, and brain volume for each diagnostic group, controlling for age, gender, and education level. Network-Based Statistics (NBS) version 1.2 [37] was used to explore correlations between FC and plasma NfL. Permutation testing with unpaired t-tests and 5000 permutations was used to determine significant results. False discovery rate (FDR) was used for multiple comparisons.

#### Longitudinal association between NfL and multi-modal neuroimaging markers

For longitudinal analyses, linear mixed effects models (LMEMs) with were used to test associations of the rate of change in plasma NfL with longitudinal data on biomarkers, incorporating fixed effects of time from baseline and random effect of each participant, with age, sex, and education years as covariances. Longitudinal changes of NfL or multi-modal neuro-imaging markers were modeled using LMEs in R with the nlme package (version 3.1) as:

$$\eqalign{{Y_{i,j}} = & {\beta _0} + {\beta _1}Tim{e_{i,j}} + {\beta _2}Grou{p_j} + {\beta _3}Tim{e_{i,j}}*Grou{p_j} \cr & + {\beta _4}Ag{e_j} + {\beta _5}Se{x_j} + {\beta _6}Ed{u_j} + {\mu _{0,j}} + {\mu _{1,j}}Tim{e_{i,j}} + { \epsilon _{i,j}} \cr}$$

Where ${Y}_{i,j}$ denotes the NfL concentration or multimodal neuro-imaging markers, ${Time}_{i,j}$ denotes the time point at time $i$ for individual $j$, ${Age}_{j}$ and ${Edu}_{j}$ denotes the mean-centered baseline age and education years for individual $j$, and ${Sex}_{j}$ denotes male or female for individual $j$. The fixed effects are described by ${\beta }_{0}$, ${\beta }_{1}$, ${\beta }_{2}$, ${\beta }_{3} ,{\beta }_{4} ,{\beta }_{5}$and ${\beta }_{6}$, whereas ${\mu }_{0,j}$ and ${\mu }_{1,j}$ denotes the random intercepts and slopes, respectively. The participant-specific slopes (${\delta }_{j}$) is estimated as ${\delta }_{j}={\beta }_{1}+{\mu }_{1,j}$. The Pearson’s correlation coefficient (r) was determined for participant-specific slope –slope (δ – δ) associations.

LMEMs is particularly robust in handling the complexities associated with longitudinal data, including attrition and intermittent missing data points, which are common in long-term studies. These methods enhanced the robustness of our findings, providing reliable insights into the progression of AD.

For FC, we calculated the change in FC and plasma NfL after 24 months as the change in FC and plasma NfL from the first acquisition (baseline). NBS was used to explore correlations between the rate of change in FC and plasma NfL.

#### Prediction of tau load and mediation analysis

Multiple linear regression was constructed to test the ability of baseline plasma NfL concentration to predict regional tau deposition in the brain after follow-up for 5–7 years. A mediation analysis was performed to statistically assess whether the effect of NfL on cognition was mediated by measured multi-modal brain MRI markers. The mediation analysis utilized baseline plasma NfL concentration as the predictor, multi-modal brain MRI markers as the mediator, and ADAS13 scores as the outcome variables.

All statistical analysis was performed using IBM SPSS Statistics version 26.0, and R programming language version 4.2.1 and Matlab 9.3, R2017B. Age, sex, and education years were included as covariates in all analyses. Except for longitudinal analysis, other analyses used Bonferroni corrections for multiple comparisons, *P*<0.05 were considered to be significant.

## Results

### Demographic and neuropsychological data

Baseline demographic and clinical characteristics by group was conducted using ANOVA and post hoc tests, or Kruskal-Wallis if the distribution data was not normal and did not satisfy the homogeneity test of variance. Table 1 provides the baseline characteristics of the ADNI sample, which includes 135 CN- individuals, 64 CN + individuals, 212 individuals with aMCI, and 106 individuals with AD. No significant age or sex differences were found among the CN-, aMCI, and AD groups (*P*>0.05). However, it was observed that the CN + group was older and had a greater proportion of males than the CN- group (age: *P* = 0.009; gender: *P* = 0.023). The aMCI and AD groups had significantly lower education and MMSE scores when compared to the CN- group (Education years: aMCI versus CN-, *P* = 0.004; AD versus CN-, *P*<0.001; MMSE scores: aMCI/AD versus CN-, *P*<0.001). Conversely, there were no significant differences between the CN + and CN- groups (*P*>0.05). Furthermore, the CSF Aβ42 and the overall mean cortical AV45 SUVR values in the CN+, aMCI, and AD groups were significantly higher than those in the CN- group (*P*<0.001) (Table 1).

**Table 1:** Characteristics of the study cohort

Group	a	b	c	d
	CN-	CN+	MCI	AD
N	135	64	212	106
Age (years)	72 ± 5.66	**75.32** ± **5.46**^a**^	73.12 ± 6.67	73.46 ± 8.36
Gender (man/female)	69/66	**22/42** ^*^	119/93	57/49
Education (years)	17.02 ± 2.49	16.34 ± 2.32	**16.03** ± **2.73**^a**^	**15.64** ± **2.64**^a***^
MMSE (0-30points)	29.09 ± 1.25	29.17 ± 0.86	**27.62** ± **1.84**^abc****^	**23.16** ± **2.05**^abc****^
CSF Aβ42 (ng/L)	234.71 ± 24.79	**137.86 ± 21.54** ^a***d*^	**135.19 ± 23.07** ^a***d**^	**126.55 ± 20.41** ^a***b*c**^
PET Aβ42 (ng/L)	0.71 ± 0.35	**0.93 ± 0.84** ^a***c***d***^	**0.98 ± 0.93** ^a***b***d****^	**1.05 ± 0.83** ^a***b***c***^
Plasma NfL (pg/mL)	29.83 ± 10.56	39.34 ± 13.86	41.92 ± 16.94	45.06 ± 17.51

Validation group	CN-	CN+		
N	49	49		
Age (years)	74.82 ± 5.17	74.80 ± 5.18		
Gender (man/female)	17/32	17/32		
Education (years)	16.88 ± 2.39	16.37 ± 2.25		
MMSE (0-30points)	29.19 ± 1.38	29.10 ± 0.97		
CSF Aβ42 (ng/L)	235.90 ± 25.99	138.86 ± 21.17***		
PET Aβ42 (ng/L)	0.70 ± 0.35	0.93 ± 0.84***		
Plasma NfL (pg/mL)	33.22 ± 11.50	38.56 ± 14.16*		

All values are indicated as mean ± standard deviation except for sex. The *P* value in the experiment group indicates the value assessed with analyses of variance (ANOVA) among Aβ − and Aβ + CN, MCI, and AD for each variable except for sex, where a contingency chi-square test was performed with Bonferroni corrections. The *P* value in the validation group indicates the value assessed with paired sample T test between CN- and CN + group. Post-hoc Bonferroni analysis provided significant differences between groups: a from CN−; b CN+; c MCI; d AD; *P value < 0.05, ** *P* value < 0.01; *** *P* value < 0.001

*Abbreviations* CN- Amyloid-beta negative cognitively normal, CN + Amyloid-beta positive cognitively normal, MCI mild cognitive impairment, AD Alzheimer’s disease, MMSE mini-mental state examination; PET, positron emission tomography; CSF, cerebrospinal fluid; NfL, neurofilament light

To avoid age and gender influence on the comparison of NfL levels between the CN- and CN + groups, a validation group comprising 49 CN + individuals and 49 age, sex, and education years matched CN- individuals were enrolled. Within the validation group of CN + and CN- groups, a paired sample T test was utilized to compare baseline demographic and clinical characteristics. There were no significant differences in age, sex, education years, or MMSE scores between the CN + and CN- groups (*P*>0.05). However, it was observed that the CSF Aβ42 and the overall mean cortical AV45 SUVR values were higher in the CN + group than in the CN- group (*P*<0.001) (Table 1).

### Plasma NfL concentration across the AD spectrum

The comparison of baseline NfL concentrations among CN-, CN+, MCI, and AD groups was conducted using ANOVA and post hoc tests. Within the validation cohort comprising CN + and CN- groups, a paired sample t-test was employed to compare baseline NfL concentrations.

**Fig. 1:**
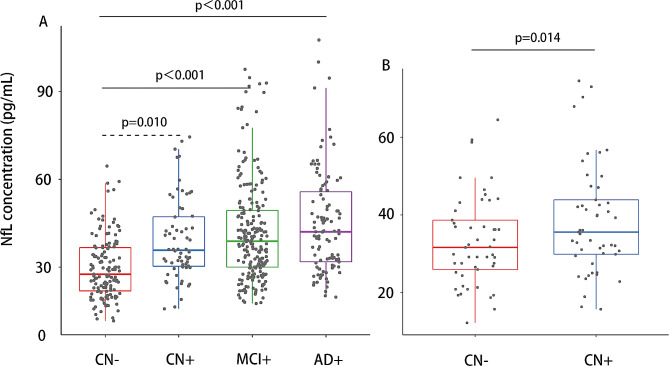
Baseline plasma NfL concentrations. Baseline plasma NfL concentrations across AD spectrum (**A**) and the validation group (**B**). Data were analyzed using ANOVA followed by Tukey post-hoc analysis. Age, sex, and education years were included as covariates in the analysis. Abbreviations: NfL, neurofilament light chain; CN-, cognitively normal participants with negative Aβ; CN+, cognitively normal participants with positive Aβ; MCI+, mild cognitive impairment patients with positive Aβ; AD+, Alzheimer’s Disease patients with positive Aβ

Results indicated significant differences in plasma NfL concentrations among the CN-, CN+, MCI, and AD groups (ANOVA with Bonferroni post hoc test; F = 23.26, *P*<0.001). The plasma NfL concentration was significantly higher in the CN+, MCI, and AD groups after adjusting for age, sex, and years of education. All differences remained significant after Bonferroni correction, except the difference between the CN- and CN + groups, which was marginally significant (*P* = 0.058). Similar results were observed in the validation group, where the plasma NfL concentration in the CN + group was significantly higher than that in the age-, sex-, and education-matched CN- group (paired t-test; *P* = 0.014) (Fig. 1).

To further validate that plasma NfL is elevated in the progression of AD, the NfL concentration in each group was compared to a normal NfL cutoff using a one-sample t-test. Results from the one-sample t-test indicated that the mean NfL concentration in the CN+, MCI, and AD groups was significantly greater than the normal cutoff of 35.02 pg/mL (NfL in CN + group = 39.34, *P* = 0.015; NfL in MCI group = 41.9, *P*<0.001; NfL in AD group = 45.06, *P*<0.001). In contrast, the mean NfL concentration in the CN- group was significantly lower than 35.02 pg/mL (NfL in CN- group = 29.83, *P*<0.001).

### Association between NfL and multi-modal neuro-imaging features

Partial correlation analysis and NBS is used to explore the relationship between baseline NfL concentration and multimodal image characteristics, with age, gender and education level as covariables. For longitudinal analyses, LMEMs with were used to test associations of the rate of change in plasma NfL with longitudinal data on biomarkers. Because NfL is related to the image features of many brain regions, if they are listed one by one, the results will be too lengthy. So, we use *P*<0.05 in the text. The correlation between specific NfL and multimodal imaging features of each brain region is listed in Supplementary Tables 1–6 for an in-depth review.

In summary, our results show that elevated baseline concentrations of NfL are associated with Aβ deposition, brain atrophy, brain connectome, glucose metabolism, and brain perfusion in AD signature regions, including the precuneus, lateral temporal cortex, inferior parietal cortex, amygdala, entorhinal gyrus, and hippocampus in aMCI patients. Further, the longitudinal analysis shows that the change rate of NfL is related to the change rate of brain atrophy in AD-characteristic brain regions. Although this tendency was not prominent in the other groups.

#### Amyloid-β pathology

In this study, all participants underwent Aβ-PET at baseline. The overall mean cortical AV45 SUVR was found to be significantly different among the CN-, CN+, aMCI, and AD groups, with a sequential increase in uptake observed across the four groups (ANOVA and Bonferroni post hoc test; F = 449.1; *P*<0.001). There was no significant correlation between plasma NfL and overall mean cortical AV45 SUVR for any of the four groups.

**Fig. 2:**
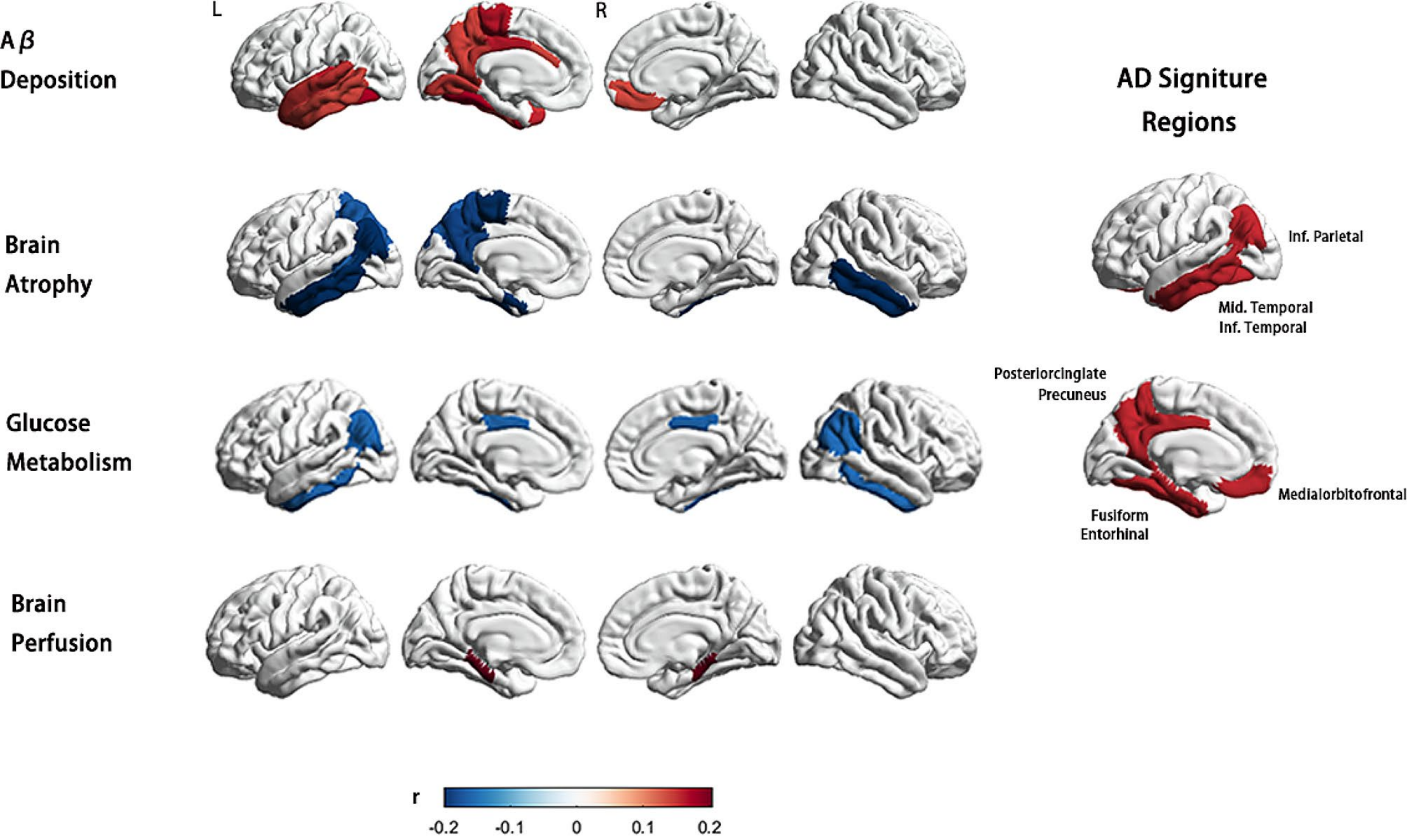
Association of baseline plasma NfL concentrations with multi-modal neuroimaging markers in the MCI group. ROI-wise partial correlations (adjusted for age, sex, and education years, *P*<0.05) between plasma NfL concentration and Aβ deposition, brain atrophy, glucose metabolism, and brain perfusion. The scale bar shows the correlation coefficient from − 0.2 to + 0.2. The elevated baseline concentrations of NfL in MCI group were associated with multi-modal neuro-imaging markers mainly in AD signature regions, including AV45 SUVR in right medial orbitofrontal cortex, left entorhinal cortex, lateral temporal cortex (including bankssts, superior temporal, inferior temporal gyrus and transverse temporal cortex), fusiform gyrus, temporopolar cortex, para-hippocampal cortex, isthmus cingulate cortex, precuneus and posterior cingulate cortex; cortical volume in the bilateral entorhinal gyrus, inferior and middle temporal gyrus, left bankssts, inferior parietal cortex, and precuneus; FDG SUVR in priori defined ROIs including bilateral angular gyrus, posterior cingulate/precuneus, and inferior temporal cortex; mean CBF in bilateral para-hippocampus. Abbreviations: L, left; R, right; r, partial correlation coefficient; NfL, neurofilament light protein; ROI, region of interest; SUVR, standardized uptake value

However, ROI-based analysis revealed that in the aMCI group, plasma NfL concentration was associated with AV45 SUVR in widespread brain areas, particularly those that are vulnerable to AD. These areas included the bilateral medial orbitofrontal cortex, left entorhinal cortex, lateral temporal cortex, fusiform gyrus, temporopolar cortex, para-hippocampal cortex, isthmus cingulate cortex, precuneus, and posterior cingulate cortex. Furthermore, plasma NfL in the aMCI group was also associated with AV45 SUVR in the left lingual gyrus, superior frontal gyrus, paracentral cortex, and rostral anterior cingulate cortex (Fig. 2).

**Fig. 3:**
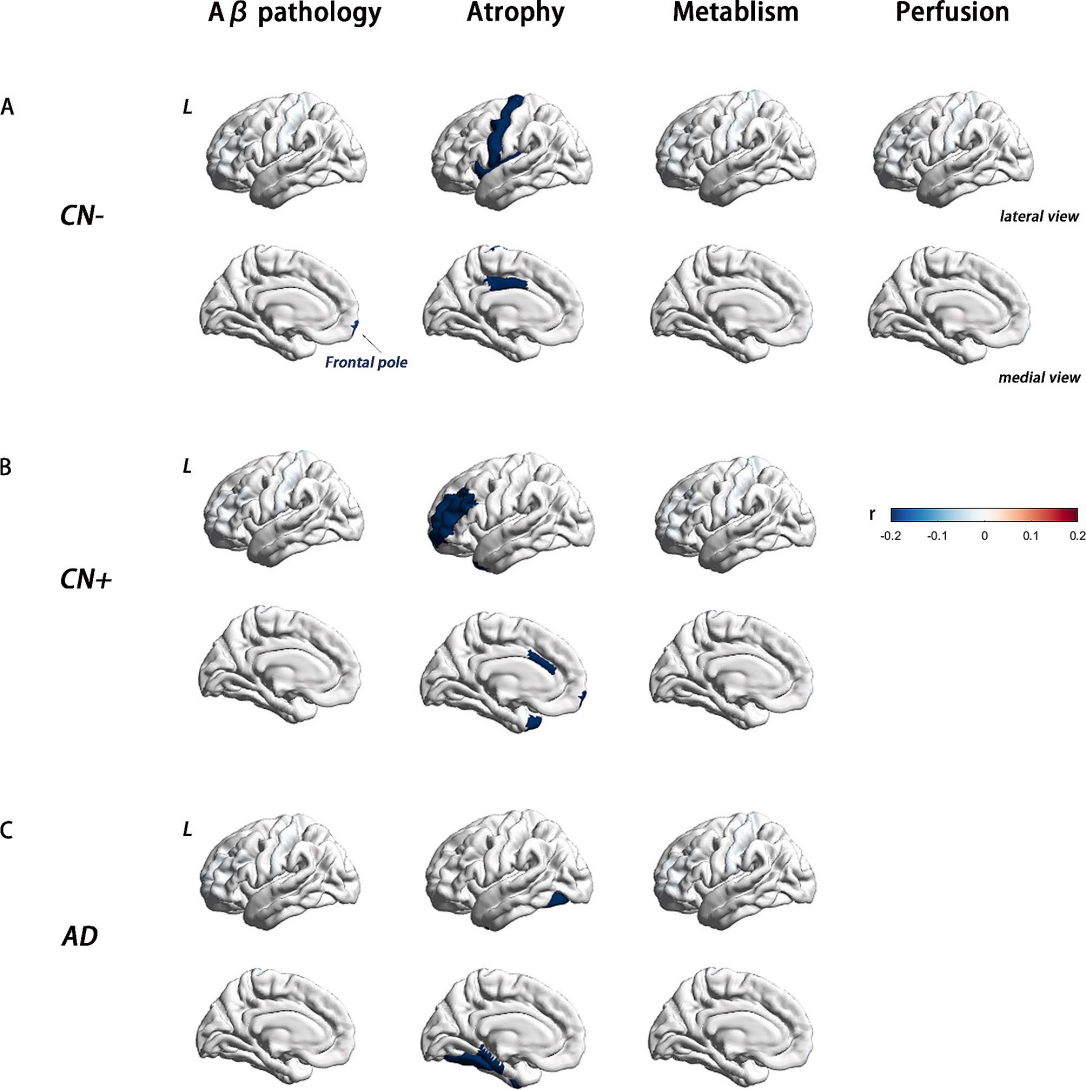
Association between baseline plasma NfL concentrations and multi-modal neuroimaging markers in CN-, CN+, and AD groups. ROI-wise partial correlations (adjusted for age, sex, and education years, *P*<0.05) between plasma NfL concentration and Aβ deposition, brain atrophy, glucose metabolism, and, brain perfusion in CN-, CN+, and AD groups. The scale bar shows the correlation coefficient from − 0.2 to + 0.2. (**A**) In the CN- group, plasma NfL was correlated with AV45 SUVR in the left frontal pole; the cortical volume of the left posterior cingulate, precentral, insula, right inferior temporal gyrus, right isthmus cingulate; mean CBF in the left pallidum. There was no significant correlation between baseline plasma NfL and the meta-ROI SUVR. (**B**) In the CN + group, there was no significant correlation between baseline plasma NfL and AV45 and FDG SUVR; The baseline plasma NfL was negatively correlated with the cortical volume in the left caudal anterior cingulate, frontal pole, rostral middle frontal gyrus, temporal pole, and right inferior parietal gyrus. (**C**) Among patients with AD, AV45 and FDG SUVR in no region were associated with plasma NfL levels; plasma NfL was significantly correlated with cortical volume in the right pericalcarine, middle temporal gyrus, and bilateral fusiform. Abbreviations: NfL, neurofilament light protein; ROI, region of interest; SUVR, standardized uptake value. FDG meta-ROI: bilateral angular gyrus, posterior cingulate/precuneus, and inferior temporal cortex

In the CN-, CN+, and AD groups, no or only a few regions were found to be associated with baseline plasma NfL concentration. Specifically, in the CN + and AD groups, no regions were associated with plasma NfL, and in the CN- group, plasma NfL was only correlated with SUVR in the left frontal pole (partial correlation *P*<0.05, age sex, and education years as covariates) (Fig. 3).

The longitudinal analysis encompassed 131 patients with aMCI who had both baseline and follow-up data. However, no region was identified as being associated with the rate of change in NfL concentration.

#### Brain metabolism

A total of 135 CN-, 64 CN+, 210 aMCI, and 105 AD participants included in the study had FDG-PET data available. Analysis revealed a group difference in mean and maximum FDG SUVR within the composite-ROI, comprising bilateral angular gyrus, posterior cingulate/precuneus, and inferior temporal cortex (mean SUVR F = 107.524, *P*<0.001; max SUVR F = 107.524, *P*<0.001). Post hoc analyses showed that AD and aMCI participants had a significantly higher overall uptake compared to control participants (corrected *P*<0.001), while no significant difference was observed between CN- and CN + groups (*P* = 0.370). Notably, a significant correlation was found between baseline NfL levels in the aMCI group and both the mean and max meta-ROI SUVR (*P*_*mean SUVR*_ value = 0.017, *r*=-0.165; *P*_max SUVR_ value = 00.024, *r*=-0.157) (Fig. 2). Conversely, no significant correlation was detected between baseline plasma NfL and the meta-ROI SUVR in the other three groups (Fig. 3).

Longitudinally, there were 103 aMCI patients with baseline and follow-up data included in the longitudinal analysis. The mean FDG SUVR was found to be associated with the rate of NfL change (*P* = 0.008, t = -2.69).

#### Brain perfusion

A total of 32 CN-, 11 CN+, 50 aMCI, and 24 AD participants underwent ASL imaging. Due to the limited number of ASL data in CN + individuals, they were combined with the aMCI cohort, resulting in a single group of 61 participants. Owing to the absence of ASL data in the majority of AD participants, this group was excluded from the analysis.

Partial correlation analyses revealed a significant positive association between baseline plasma levels of NfL and CBF in bilateral para-hippocampal regions, as well as in the left entorhinal gyrus and hippocampus, in the CN + and aMCI groups (Fig. 2). In order to verify the robustness of the findings, we re-examined this correlation solely in the aMCI group, where similar results were obtained. Specifically, a positive correlation trend between NfL and CBF was observed in bilateral para-hippocampal regions. By contrast, in the CN- group, the baseline plasma NfL presented a significant positive correlation with CBF only in the left pallidum (Fig. 3) (partial correlation *P*<0.05, age sex, and education years as covariates).

#### Brain atrophy

The present study utilized 91 CN-, 66 CN+, 145 aMCI, and 59 AD participants with excellent overall segmentation T1-weighted images to investigate the relationship between plasma NfL and brain atrophy in AD.

The results showed that in the aMCI group, plasma NfL was primarily associated with cortical and subcortical volume in AD signature areas, including the bilateral amygdala, hippocampus, inferior and middle temporal gyrus, left bankssts, inferior parietal cortex, entorhinal gyrus and precuneus. These associations remained significant even after controlling for age, sex, and education years as covariates (Fig. 2). Interestingly, the pattern of association between plasma NfL and regional atrophy differed between aMCI and the other three groups. In the CN- group, NfL-related brain regions were mostly observed in frontal areas, with limited involvement of the temporal lobe. Specifically, plasma NfL was correlated with the cortical volume of the left posterior cingulate, precentral, insula, right inferior temporal gyrus, and right isthmus cingulate, as well as the bilateral hippocampus and amygdala. In CN + patients, plasma NfL was correlated with the cortical volume of the left caudal anterior cingulate, frontal pole, rostral middle frontal gyrus, temporal pole, and right inferior parietal gyrus. On the other hand, in the AD group, the NfL-related brain regions were fewer and more scattered, spanning across different brain regions. In AD patients, plasma NfL was significantly associated with cortical volume in the right pericalcarine, middle temporal gyrus, and bilateral fusiform (Fig. 3).

Furthermore, to investigate the hippocampus in more detail, it was divided into seven subregions: the subiculum complex (anterior hippocampus), the CA subregions comprising CA1–4 (posterior regions), the DG, and the hippocampal fissure. The partial correlation results demonstrated that plasma NfL was significantly correlated with cortical volume in bilateral CA1, CA2_3, CA4_DG, fimbria, presubiculum, and subiculum among aMCI patients. The correlation between NfL and pre-subiculum and subiculum was particularly strong and significant. In contrast, the plasma NfL was significantly correlated with CA2_3, CA4_DG, and hippocampal fissure (Table 2).

**Table 2:** Association between plasma NfL and hippocampus subfields volume

Hippocampus subfields	Correlation *P* value (*r*)	
	CN-	MCI
CA1	0.063 (-0.199)	**0.001 (-0.267)**
CA2_3	**0.032 (-0.228)**	**0.007 (-0.224)**
CA4_DG	**0.028 (-0.235)**	**0.004 (-0.241)**
Fimbria	0.416 (0.088)	**0.006 (-0.231)**
Hippocampal Fissure	**0.021 (-0.246)**	0.145 (-0.123)
Presubiculum	0.327 (-0.106)	**<0.001 (-0.327)**
Subiculum	0.066 (-0.197)	**<0.001 (-0.282)**
Tail	0.397 (-0.091)	0.052 (-0.164)

Partial correlation results showed that plasma NfL was closely related to cortical volume in bilateral CA1, CA2_3, CA4_DG, fimbria, presubiculum, and subiculum in patients with MCI. The correlation between NfL and pre-subiculum and subiculum was particularly pronounced and significant. In CN- group, plasma NfL was significantly correlated with CA2_3, CA4_DG, and hippocampal fissure in the CN- group. No correlation between plasma NfL and hippocampal subregion volume was found in the CN + and AD groups. The composite-ROI includes the bilateral angular gyrus, posterior cingulate/precuneus, and inferior temporal cortex

*Abbreviations* CA, cornuammonis; DG, dentate gyrus. CN- Amyloid-beta negative cognitively normal, CN + Amyloid-beta positive cognitively normal, MCI mild cognitive impairment, AD Alzheimer’s disease

There were 169 aMCI patients with the baseline and follow-up data for longitudinal analysis. Results from LMEMs revealed significant associations between the rate of change in plasma NfL and cortical volume of bilateral inferior parietal cortex, inferior temporal cortex, lateral occipital cortex, left lateral orbitofrontal cortex, middle temporal cortex, superior frontal cortex, parahippocampal gyrus, and right frontal pole. Additionally, it was correlated with subcortical volume of bilateral hippocampus and right accumbens area. For the Hippocampal subfields, it was associated with volume of right CA2_3, CA4_DG, presubiculum, and subiculum.

#### Brain connectomes

This study investigated the relationship between plasma NfL and brain connectomes by utilizing both structural and FC measures, using DTI and fMRI, respectively. In terms of structural connectivity, the study involved 35 CN-, 14 CN+, 49 aMCI, and 31 AD participants who underwent DTI examination. Due to the limited availability of DTI data for CN + participants, these individuals were combined with aMCI participants into a single group. Analysis of this combined group revealed that white matter fibers associated with elevated plasma NfL levels were primarily association fibers that connected different regions of the brain that are vulnerable to AD. Specifically, plasma NfL levels were found to be significantly positively correlated with AD, MD, and RD values of the right cingulum, right uncinate fasciculus, and left fornix, and negatively correlated with FA values of the left cingulum and uncinate fasciculus, controlling for age, sex, and education years as covariates. To address potential confounding effects resulting from the inclusion of CN + and aMCI participants in a single group, the analysis was replicated using only aMCI patients, which yielded similar results. In contrast, analysis of AD patients revealed that elevated plasma NfL was correlated with injury in multiple projection fibers, including the bilateral anterior and posterior limb of the internal capsule, corticospinal tract, and inferior cerebellar peduncle.

Besides, our findings demonstrated a significant association between the rate of change in plasma NfL concentration and disrupted WM microstructure across the brain. Specifically, correlations were observed with AD, MD, and RD values in several brain regions. These included the bilateral corticospinal tract, cerebral peduncle, and left inferior cerebellar peduncle, superior cerebellar peduncle, superior fronto-occipital fasciculus, and right medial lemniscus, with AD and MD values in the right cerebral peduncle; MD and RD values in the left medial lemniscus and posterior thalamic radiation; AD values in the left superior cerebellar peduncle and tapetum; MD values in the right cingulum; and RD values in the bilateral splenium of the corpus callosum, the entire corpus callosum, the left external capsule, the splenium of the corpus callosum, and the right anterior limb of the internal capsule, and sagittal stratum. Furthermore, significant associations were found with FA in the left corticospinal tract, anterior limb of the internal capsule, posterior limb of the internal capsule, posterior thalamic radiation, superior longitudinal fasciculus, the splenium of the corpus callosum, and bilateral splenium of the corpus callosum. Specific p values are provided in Supplementary Table 6.

**Fig. 4:**
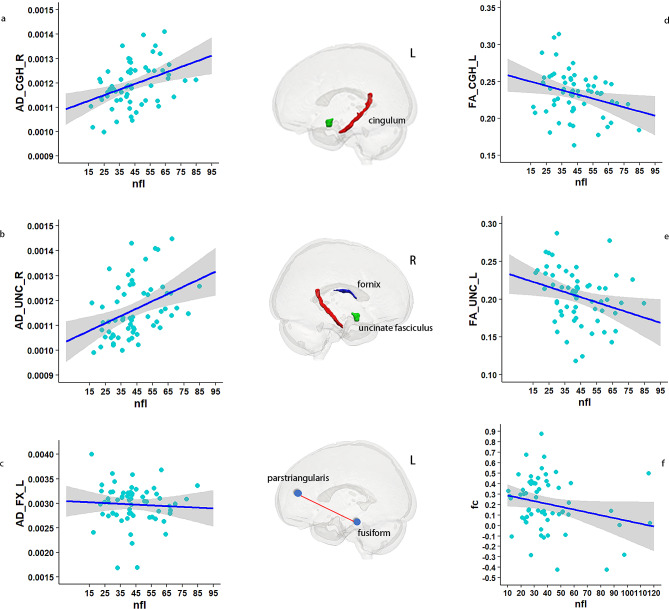
Correlation of plasma NfL with brain connectome in MCI (*n* = 49). The provided figures (**a**-**e**) visually depict the relationship between plasma NfL and the axial diffusivity of specific brain tracts, namely, the right cingulum, right uncinate fasciculus, left fornix, and the fractional anisotropy of the left cingulum and uncinate fasciculus. Additionally, figure **f** illustrates the functional connectivity between the left fusiform and parstriangularis regions, which displayed a negative correlation with plasma NfL (*P*<0.05). The shaded region surrounding the linear fit line in the figures represents one standard error of the mean, as determined by the LME model. Lastly, the middle image portrays the structural and functional tracts that exhibit a significant correlation with plasma NfL. Abbreviations: AD_CGH_R, axial diffusivity of right cingulum; AD_UNC_R, axial diffusivity of right uncinate fasciculus; AD_FX_L, axial diffusivity of left fornix; FA_CGH_L; FA_CGH_L, fractional anisotropy of left cingulum; FA_UNC_L, fractional anisotropy of left uncinate fasciculus; fc, functional connectivity

As for FC, 62 aMCI participants had baseline resting-state fMRI data. The study found a significant positive correlation between plasma NfL levels and FC between the left fusiform and parstriangularis regions in the aMCI group (*P*<0.05, Fig. 4). Moreover, our longitudinal analysis of 37 aMCI patients with the baseline and follow-up at year 2 data demonstrated that the rate of change of FC between the left entorhinal and transverse temporal gyrus, right precuneus and right parsopercularis, as well as left inferior temporal gyrus and rostral-anterior cingulate, was significantly correlated with the rate of change of plasma NfL.

### Prediction of tau load by baseline plasma NfL concentrations

**Fig. 5:**
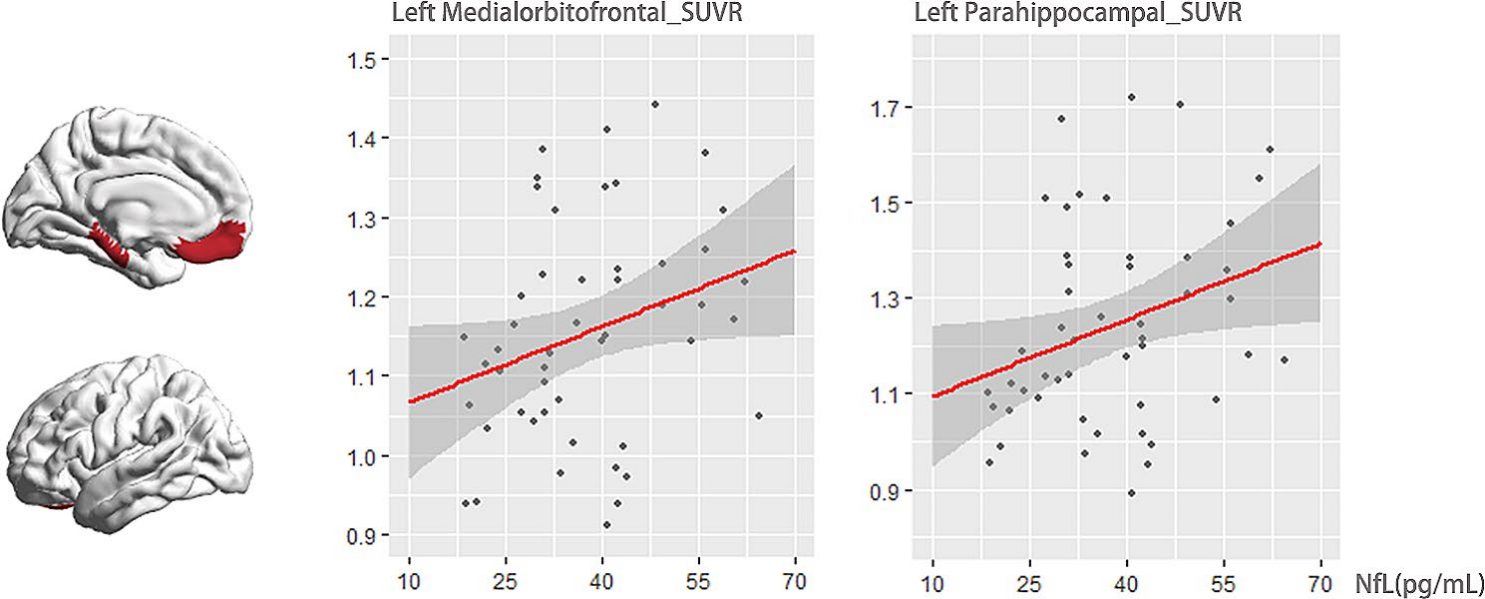
Association of plasma NfL with flortaucipir uptake after approximately 5 to 7 years. Among the 50 Aβ + CN and MCI participants with tau PET data available, multivariable linear regression analysis was performed to explore the predictive value of baseline plasma NfL concentrations for future tau deposition in the brain. Regional tau burden after 5 to 7 years of follow-up was used as the dependent variable and baseline NfL as a predictor, controlling for age, sex, and years of education. The baseline NfL concentration and SUVR PET uptake in the left medial orbitofrontal cortex and para-hippocampus were positively correlated (F = 2.474, *P*<0.029; F = 2.224, *P*<0.042). Abbreviations: NfL, neurofilament light protein; SUVR, standardized uptake value

Multiple linear regression was conducted to investigate the potential predictive value of baseline plasma concentrations of NfL in relation to future tau deposition in the brain. A total of 50 participants diagnosed with CN + and aMCI were assessed for tau PET data. A multivariable linear regression analysis was conducted to investigate the potential predictive value of baseline plasma concentrations of NfL in relation to future tau deposition in the brain. The dependent variable for this analysis was regional tau burden after a 5 to 7 years follow-up, with baseline NfL serving as the independent predictor. Age, gender, and education years were controlled for in the analysis. The findings revealed that baseline NfL had a significant effect in predicting increased tau burden in the left medial orbitofrontal cortex and para-hippocampus (β = 0.336, *P* = 0.032; β = 0.313, *P* = 0.047) (Fig. 5).

### Mediation analysis

Mediation analysis was sought to determine whether the relationship between plasma NfL levels and cognitive performance is mediated by multi-dimensional brain abnormalities. We specifically examined neuroimaging features that showed significant associations with both baseline NfL concentrations and Alzheimer’s Disease Assessment Scale (ADAS) scores. The results revealed that increased atrophy in the left middle and inferior temporal gyri partially mediated the impact of plasma NfL on cognitive function. Furthermore, reductions in both mean and maximum glucose metabolism across a composite ROI, as well as changes in FA, MD, and RD values in the cingulum, were significant mediators. Bootstrapping methods confirmed these findings; non-parametric bootstrap analysis dictates that a 95% confidence interval excluding zero indicates statistical significance.

**Fig. 6:**
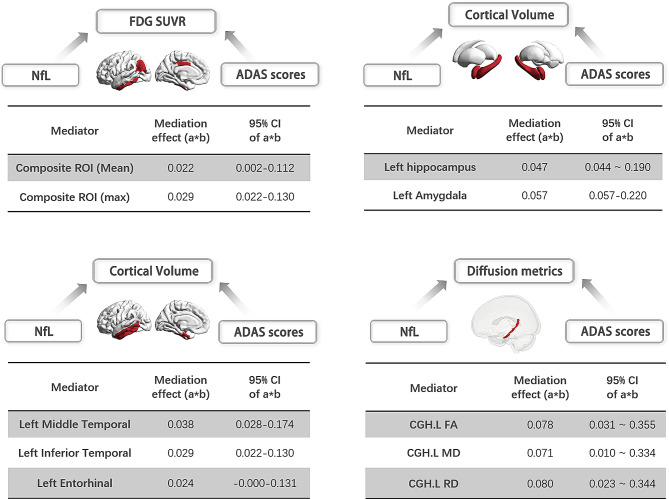
Relationships between plasma NfL, multi-modal brain MRI markers, and cognition were revealed in MCI patients. There was a strong effect of plasma NFL on general cognition mediated by the decreased mean and maximum FDG SUVR in the composite-ROI (mediation effect = 0.022 [0.002‒0.112]; 0.029 [0.022‒0.130]); brain atrophy in the cortical left middle temporal gyrus, inferior temporal gyrus, entorhinal (mediation effect = 0.038 [0.028‒0.174]; 0.029 [0.002‒0.144]; 0.024 [0.000‒0.131]) and subcortical structures include left hippocampus and amygdala (mediation effect = 0.047 [0.044‒0.190]; 0.057 [0.057‒0.220]); the FA, MD, and RD values of the cingulum (mediation effect = 0.078 [0.031‒0.355]; 0.071 [0.010‒0.334]; 0.080 [0.023‒0.344]). Abbreviations: NfL, neurofilament light protein; ADAS13, Alzheimer’s Disease Assessment Scale Cognition 13-item scale; Composite-ROI includes the bilateral angular gyrus, posterior cingulate/precuneus, and inferior temporal cortex; CGH, cingulum; FA, fractional anisotropy; MD, mean diffusivity; RD, radial diffusivity

Specifically, the indirect effects included: mean FDG SUVR in the composite ROI was 0.029 (95% CI [0.022–0.130]); maximum FDG SUVR in the composite ROI was 0.022 (95% CI [0.002–0.112]); cortical volumes in the left entorhinal cortex at 0.024 (95% CI [-0.000, 0.131]), left inferior temporal gyrus at 0.029 (95% CI [0.002, 0.144]), left middle temporal gyrus at 0.038 (95% CI [0.028, 0.174]), amygdala at 0.057 (95% CI [0.057–0.220]), and hippocampus at 0.047 (95% CI [0.044–0.190]). The indirect effects for FA, MD, and RD in the cingulum were 0.078 (95% CI [0.031–0.355]), 0.071 (95% CI [0.010–0.334]), and 0.080 (95% CI [0.023–0.344]), respectively (Fig. 6).

## Discussion

The concentration of NfL in blood has shown promise as a potential biomarker for the diagnosis and prognosis of AD. However, the extent to which NfL is associated with multi-modal neuroimaging features and its ability to predict future tau deposition has not been thoroughly researched. Our study aims to address these gaps by revealing the following findings: [1] elevated baseline concentrations and change rate of NfL in individuals with aMCI were strongly associated with Aβ deposition, brain atrophy, brain connectome, glucose metabolism, and brain perfusion in AD signature regions [2]. Baseline NfL showed strong predictive value for increasing tau burden in the medial orbitofrontal cortex and para-hippocampal regions in both the CN + and the aMCI groups [3]. The multi-modal neuro-imaging features mediated the association between plasma NfL and cognitive performance.

Plasma NfL has emerged as a promising biomarker for AD research due to its cost-effectiveness and superior tolerability compared to other biomarker measures such as MRI, PET, or CSF biomarkers. Although previous studies have focused on the clinical utility of plasma NfL for differentiating AD and aMCI patients from controls, fewer have investigated its potential as a preclinical biomarker for early disease diagnosis. A previous study compared CN + and CN- participants and found an abnormally high concentration of plasma NfL and its rate of change [38]. Our study further extends their findings to encompass the entire AD spectrum, including CN-, CN+, Aβ positive aMCI, and Aβ positive AD groups. We found that baseline NfL concentration was higher in CN+, aMCI, and AD groups compared to the CN- group, reinforcing the potential of NfL as a valuable biomarker for improving diagnostic and prognostic accuracy in AD patients.

The findings of our study demonstrate a strong association between elevated baseline concentrations of NfL in individuals with aMCI and several key markers of AD, including Aβ deposition, brain atrophy, brain connectome, glucose metabolism, and brain perfusion in regions that are characteristic of AD. Additionally, changes in NfL levels were significantly linked to changes in brain thickness in regions characteristic of AD. The findings of this study are consistent with previous research in this area. For instance, Yi Chen et al. found that plasma NfL levels were significantly elevated and related to hippocampal atrophy, larger ventricular volume, and baseline FDG SUVRs in various brain regions in aMCI group [39]. Similarly, Mattsson N et al. observed a correlation between high plasma NfL and AD-related atrophy and brain hypometabolism in participants with aMCI [25]. In addition, a study focused on amyloid-positive cognitively impaired individuals (clinically defined as having aMCI or AD dementia) found that higher concentrations of plasma and cerebrospinal fluid NfL were associated with hypometabolism in AD-vulnerable regions at baseline and longitudinally [40]. Regarding brain structural connectivity, Nabizadeh F demonstrated a significant association between plasma NfL levels and disrupted WM microstructure across the brain in distinct areas [15], which overlapped with the present study’s findings. Specifically, higher plasma NfL was related to lower FA and higher RD, AD, and MD in the fornix, uncinate fasciculus, and hippocampal cingulum. For FC, a recently published article revealed that plasma NfL was positively correlated with the deterioration of FC within the default mode network in autosomal dominant AD mutation carriers [41].

However, some studies have found results inconsistent with ours, where no cross-sectional associations were observed between NfL and any neuroimaging measures in 79 participants with aMCI [42]. We suspect that this inconsistency may be due to the lack of further classification of aMCI, which includes simple memory impairment and memory with other impairments. In our study, we selectively included those with simple memory impairment and excluded those with negative Aβ protein, who are more likely to be in the prodromal stage of AD and reflect the characteristic changes of AD. Additionally, although previous studies found the change in plasma NfL to be associated with the change in global cognition, attention, hippocampal atrophy, and amyloid PET [42–44], our results only found a significant association between the rate of NfL change and the change in cortical atrophy in some brain region. This may be due to the limited availability of NfL data, which only covered three-time points for most patients. Future analysis at more time points is required to reduce data bias and confirm these findings. Furthermore, our study provides the first evidence for the correlation between brain perfusion and plasma NfL, suggesting that reduced brain perfusion in aMCI patients may cause damage to axons of neurons, ultimately resulting in elevated NfL levels in the blood. Overall, our results support an association between plasma NfL and multi-modal neuroimaging features in AD-vulnerable regions, providing insight into NfL as a potential biomarker for tracking disease progression and facilitating its proper use in AD research and therapeutic trials.

Secondly, our study has revealed that baseline NfL levels in CN + and aMCI participants have a significant predictive value in elevating tau burden in the left medial orbitofrontal cortex and para-hippocampus. It is noteworthy that prior research exploring the relationship between NfL and tau pathology in AD has primarily focused on CSF, post-mortem tissue, and blood [45–48]. Specifically, studies have demonstrated that elevated NfL levels in blood are associated with increased total and phosphorylated tau levels in symptomatic carriers of an ADAD mutation and greater neurofibrillary tangles in post-mortem tissue of older adults with AD dementia, but not plasma tau [49]. To our knowledge, only a limited number of studies have investigated the link between plasma NfL levels and PET tau in AD. Recently, one such study demonstrated that in non-demented Presenilin-1 *(PSEN1)* E280A mutation carriers, higher plasma NfL levels were linked to greater tau burden in regions such as the precuneus and temporal lobe, including the entorhinal cortex [50]. Notably, the regions identified in our study differ from those found in the aforementioned research, which may be attributed to our sample consisting of individuals with autosomal-dominant AD rather than sporadic AD. Nonetheless, our research, combined with prior investigations, highlights a possible relationship between plasma NfL and aggregated neurofibrillary tangles measured by [F18] FTP PET. Longitudinal data will be required to better address whether plasma NfL has the potential to be an effective predictor of downstream tau pathology.

NfL is released into the CSF and subsequently the plasma primarily due to axonal damage. Given that axonal injury is a hallmark of AD neurodegeneration, elevated plasma NfL levels are thought to reflect ongoing neurodegenerative processes [51]. This association provides a plausible link to neuroimaging findings showing brain atrophy, particularly in regions vulnerable to AD pathology such as the medial temporal lobe. Besides, NfL may be the downstream neurodegeneration resulting from the pathological accumulation of tau and amyloid-beta. Lastly, we also consider that the inflammatory processes or the synaptic dysfunction may relate to the mechanism underlying the correlation between plasma NfL levels and AD-related neuroimaging features [51–53].

Finally, we investigated whether the relationship between plasma levels of NfL and cognitive performance in AD was mediated by neuroimaging features in AD signature regions. Our results demonstrated that Aβ deposition and brain atrophy in the left middle and inferior temporal gyrus, glucose metabolism in the composite region of interest, as well as the RD, MD, and FA values of the cingulum, partially mediated the association between NfL levels and cognitive function. While previous studies have established a correlation between elevated NfL concentration and poor cognitive outcomes, few have explored the underlying mechanisms that link reduced neuronal integrity, as indicated by abnormal NfL levels, to cognitive function. Our findings are in concordance with the work of Min Su Kang et al. [38], who found that the association of NfL concentration with grey matter density was influenced by Aβ deposits in AD-vulnerable regions in Aβ + aMCI and AD. Likewise, Weina Yao et al. [54] reported that the effects of plasma NfL on global cognition and episodic memory in AD-spectrum patients were mediated by the functional role of several brain regions. However, these studies did not examine the mediating role of glucose metabolism, structural connectivity, and Aβ deposition. Taken together, our results point to the complex interplay between plasma NfL and multiple pathological changes that give rise to cognitive impairment in AD.

The ATN framework provides a biological construct for AD diagnosis, categorizing biomarkers into three binary categories: A for amyloid-beta deposition, T for pathologic tau, and N for neurodegeneration or neuronal injury. Our study contributes to this framework by demonstrating that plasma NfL levels serve not only as a marker of neurodegeneration (the ‘N’ in ATN) but also offer predictive insights into the other ATN categories. Elevated plasma NfL levels in our participant cohorts, particularly in cognitively normal individuals with positive amyloid-beta deposition and those with aMCI, underscore its utility in indicating early neurodegenerative changes before significant cognitive symptoms emerge. This aligns with the ‘N’ component of the ATN framework and suggests that plasma NfL could serve as a non-invasive, accessible measure of neurodegeneration across the AD spectrum.

Furthermore, our findings reveal that plasma NfL levels have a significant predictive value for future tau deposition in critical brain regions associated with AD. This ties the plasma NfL levels not just to the ‘N’ component but also provides a bridge to the ‘T’ tau pathology, indicating potential for early prognostic assessments even before significant tau pathology becomes detectable through current imaging techniques. Incorporating plasma NfL into the ATN framework could enhance its diagnostic precision by providing a more comprehensive picture of AD pathology from an earlier stage. Clinicians might leverage this information to devise personalized management plans geared towards slowing disease progression through interventions tailored to the specific biomarker profile of a patient. For instance, identifying individuals with elevated NfL levels yet minimal cognitive impairment could prioritize them for interventions aimed at mitigating further neurodegenerative changes or tau pathology development. Moreover, as part of routine clinical practice, monitoring changes in plasma NfL levels alongside other ATN biomarkers could offer insights into disease trajectory and treatment efficacy, allowing for timely adjustments to therapeutic approaches.

## Limitations and future research

The present study has certain limitations that must be acknowledged. Firstly, we acknowledge the relatively small sample size, particularly of CN + participants, in some MRI model analyses, including ASL, DTI, and brain fMRI. A larger sample size is required to better comprehend the association between plasma NfL and AD-related neuroimaging measures. Secondly, we recognize that elevated plasma NfL concentrations as a non-specific biomarker have also been observed in other neurodegenerative diseases, such as frontotemporal dementia [11] and cerebral small vessel diseases [55]. Thus, future research should include more neurodegenerative diseases to investigate the distinct roles of plasma NfL in different neurodegenerative disorders. Finally, it is important to note that we did not apply corrections for multiple comparisons in our cross-sectional association analysis. As a result, there is an increased likelihood of Type I errors, and reported p-values may reflect false positives. Therefore, any significant findings should be viewed as preliminary and in need of validation. Future studies with larger sample sizes and appropriate corrections for multiple comparisons will be essential to confirm these results and ensure their reliability.

our study also possesses noteworthy strengths. To the best of our knowledge, this represents the most extensive analysis of the correlation between NfL and other imaging biomarkers Particularly, the association between plasma NfL and brain structural, FC, and perfusion has scarcely been examined in previous studies. Through this comprehensive perspective, it is possible to gain a more profound understanding of how degeneration impacts plasma NfL concentrations. Furthermore, previous studies have primarily focused on establishing the association between NfL concentrations and imaging markers in pre-defined regions typically affected by AD. Our analysis of the relationship between NfL concentration and multidimensional neurodegeneration markers across the entire brain enabled us to gain a greater understanding of whether the levels of NfL are driven by AD-vulnerable regional neuronal injury or age-related neurodegeneration. Lastly, our study also examined the relationship between plasma NfL and PET tau load, which has previously been rarely explored.

## Conclusion

In conclusion, our study underlines the importance of NfL as a biomarker in the context of AD. The demonstrated associations between elevated plasma NfL levels and various neuroimaging markers across the AD spectrum, along with its ability to predict future tau pathology, suggest that NfL holds significant promise for early detection, diagnosis, and monitoring of this debilitating condition. The findings reinforce the applicability of NfL as a non-invasive, easily accessible biomarker that can potentially improve the precision of AD diagnoses, refine the assessment of disease progression, and aid in the tailoring of therapeutic interventions at stages where they may have the most substantial impact. Moreover, the utility of NfL may extend to a broader neurological context, serving as an indicator of central nervous system integrity in other conditions, albeit with the caveat that specificity can be improved through combinatory approaches with other disease-specific biomarkers.

The potential for NfL to revolutionize our approach to AD is evident from our study, providing a beacon of hope for improving patient outcomes via enhanced diagnostic and therapeutic strategies. Further research will build upon our findings to refine the clinical deployment of NfL and to fully unleash its prognostic and diagnostic power, thereby shaping the future landscape of neurodegenerative disease management.

### Electronic supplementary material

Below is the link to the electronic supplementary material.

Supplementary Material 1

## Data Availability

The dataset supporting the conclusions of this article is available in a publicly available repository with open access. All ADNI data have been deposited in a publicly accessible repository and can be conveniently accessed at https://adni.loni.usc.edu/.

## Abbreviations

NfL: Neurofilament light
AD: Alzheimer’s disease
MRI: Magnetic resonance imaging
DTI: Diffusion tensor imaging
PET: Positron emission tomography
fMRI: functional magnetic resonance imaging
ASL: Arterial spin labeling
Aβ: Amyloidβ
aMCI: mild cognitive impairment
CSF: Cerebrospinal fluid
ADNI: Alzheimer’s Disease Neuroimaging Initiative
CN: Cognitively normal
aMCI: amnestic mild cognitive impairment
MMSE: Mini-Mental State Examination score
CDR: Clinical Dementia Rating
SUVR: Standardized uptake value ratio
18F-FDG-PET: 18-Fluorodeoxyglucose positron emission tomography
FA: Fractional anisotropy
MD: Mean diffusivity
AD: Axial diffusion
RD: Radial diffusion
WM: White matter
ROI: Regions of interest
rs-fmri: rest-state functional MRI
QC: Quality control
AV-45: 18 F-florbetapir
CBF: Cerebral blood flow
AV-1451: Flortaucipir
ADAS13: Alzheimer’s Disease Assessment Scale Cognition 13-item scale
ANOVA: Analysis of variance
CA: Cornuammonis
DG: Dentate gyrus
