# Supplementary material for: Elevated plasma neurofilament light was associated with multi-modal neuroimaging features in Alzheimer’s disease signature regions and predicted future tau deposition
Source: BMC Neurol. 2024 Jul 6;24:236. doi: 10.1186/s12883-024-03728-7 (PMC11227162; doi:10.1186/s12883-024-03728-7)
Supplement: Supplementary file 1 — Supplementary Material 1 [file 12883_2024_3728_MOESM1_ESM.docx]

**Supplementary materials**

**Supplementary Methods**

The visual quality control includes four outcomes: Pass, Fail, Hippocampus-Only, and Partial. Pass indicates a good overall segmentation. Fail indicates a global failure due to extremely poor image quality, registration issues, or gross misestimation of the hippocampus. Fail can also indicate a processing error. Hippocampus-Only indicates a global failure of the segmentation but the hippocampi are properly estimated. Partial indicates a “failure” in one or more of 8 regions listed below. These 8 regions consist of several structures that are the most common sites of poor FreeSurfer segmentation. In cases of partial region “failures” ALL cortical regions included within that regional definition are excluded from the volumetric report regardless of which specific cortical areas were affected. For example, if a scan is rated Parietal ‘fail’ then the following sub-regions are excluded from the report because one or more of these sub-regions did not satisfy the QC requirements: Postcentral, superior parietal, paracentral, supramarginal, and inferior parietal. Subjects with an overall rating of “partial” still have usable hippocampal volume data, even when the failure occurs in the Temporal region. Most subjects will receive an outcome of pass, partial, or hippocampus only. A fail in any of the 8 regions (Frontal, Temporal, Insula, Parietal, Occipital, Cerebral WM, or Basal Ganglia) indicates that one or more of the structures in that region did not meet our QC standards.

**Supplementary Figures**


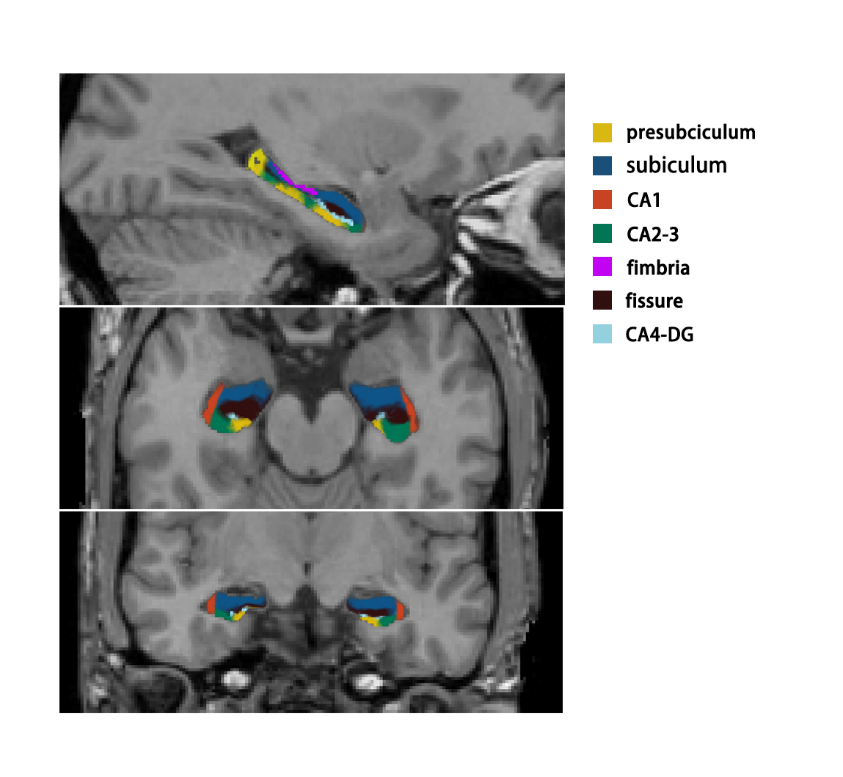


**Supplementary Figure.1 An example of hippocampal subfield segmentation via Free Surfer (from one subject).** Transverse, coronal and sagittal T1 images of hippocampal subfield segmentation via FreeSurfer, version 5.1.

Abbreviations: CA, cornu ammonis; DG, dentate gyrus.

**Supplementary Tables**

**Supplementary Table1. Associations between NfL and Aβ deposition**

|  | |  | |
| --- | --- | --- | --- |
| LH_Bankssts_SUVR | R-value | | 0.142 |
|  | P-value (two-tail) | | 0.040 |
| LH_Caudalanteriorcingulate_SUVR | R-value | | 0.137 |
|  | P-value (two-tail) | | 0.048 |
| LH_Entorhinal_SUVR | R-value | | 0.139 |
|  | P-value (two-tail) | | 0.044 |
| LH_Fusiform_SUVR | R-value | | 0.177 |
|  | P-value (two-tail) | | 0.010 |
| LH_Inferiortemporal_SUVR | R-value | | 0.139 |
|  | P-value (two-tail) | | 0.045 |
| LH_Lingual_SUVR | R-value | | 0.144 |
|  | P-value (two-tail) | | 0.037 |
| LH_Medialorbitofrontal_SUVR | R-value | | 0.170 |
|  | P-value (two-tail) | | 0.014 |
| LH_Middletemporal_SUVR | R-value | | 0.137 |
|  | P-value (two-tail) | | 0.048 |
| LH_Paracentral_SUVR | R-value | | 0.165 |
|  | P-value (two-tail) | | 0.017 |
| LH_Posteriorcingulate_SUVR | R-value | | 0.163 |
|  | P-value (two-tail) | | 0.018 |
| LH_Precuneus_SUVR | R-value | | 0.141 |
|  | P-value (two-tail) | | 0.042 |
| LH_Superiorfrontal_SUVR | R-value | | 0.148 |
|  | P-value (two-tail) | | 0.032 |
| LH_Superiortemporal_SUVR | R-value | | 0.147 |
|  | P-value (two-tail) | | 0.033 |
| LH_Temporalpole_SUVR | R-value | | 0.157 |
|  | P-value (two-tail) | | 0.023 |
| LH_Transversetemporal_SUVR | R-value | | 0.165 |
|  | P-value (two-tail) | | 0.017 |
| RH_Medialorbitofrontal_SUVR | R-value | | 0.137 |
|  | P-value (two-tail) | | 0.047 |

Notes: Partial correlation analysis is used to explore the relationship between baseline NfL concentration and Aβ deposition. The coefficient of partial correlation, denoted as R-value, measures the strength and direction of a linear relationship between baseline NfL concentration and Aβ deposition, while controlling for the effect of age, sex and education years.

Abbreviations: LH, Left Hemisphere; SUVR, standardized uptake value ratio.

**Supplementary Table2. Associations between NfL and DTI metrics**

| DTI metrics |  | |
| --- | --- | --- |
| FA_CGH_L | R-value | -0.260 |
|  | P-value (two-tail) | 0.047 |
| FA_UNC_L | R-value | -0.275 |
|  | P-value (two-tail) | 0.035 |
| MD_CGH_R | R-value | 0.326 |
|  | P-value (two-tail) | 0.012 |
| MD_UNC_R | R-value | 0.297 |
|  | P-value (two-tail) | 0.023 |
| MD_FX_L | R-value | -0.280 |
|  | P-value (two-tail) | 0.032 |
| RD_CGH_R | R-value | 0.335 |
|  | P-value (two-tail) | 0.009 |
| RD_UNC_R | R-value | 0.300 |
|  | P-value (two-tail) | 0.021 |
| RD_FX_L | R-value | -0.260 |
|  | P-value (two-tail) | 0.047 |
| AD_CGH_R | R-value | 0.274 |
|  | P-value (two-tail) | 0.036 |
| AD_UNC_R | R-value | 0.268 |
|  | P-value (two-tail) | 0.040 |
| AD_FX_L | R-value | -0.303 |
|  | P-value (two-tail) | 0.019 |

Notes: Partial correlation analysis is used to explore the relationship between baseline NfL concentration and Aβ deposition. The coefficient of partial correlation, denoted as R-value, measures the strength and direction of a linear relationship between baseline NfL concentration and Aβ deposition, while controlling for the effect of age, sex and education years.

Abbreviations: FA_CGH_L, fractional anisotropy of left cingulum; FA_UNC_L, fractional anisotropy of left uncinate fasciculus; MD_CGH_R, mean diffusivity of right cingulum; MD_UNC_R, mean diffusivity of right uncinate fasciculus; MD_FX_L, mean diffusivity of left fornix; RD_CGH_R, radial diffusion of right cingulum; RD_UNC_R, radial diffusion of right uncinate fasciculus; RD_FX_L, radial diffusion of left fornix; AD_CGH_R, axial diffusion of right cingulum; AD_UNC_R, axial diffusion of right uncinate fasciculus; AD_FX_L, axial diffusion of left fornix.

**Supplementary Table.3 Associations between NfL and FDG metrics**

| FDG metrics | |  | |
| --- | --- | --- | --- |
| Mean FDG SUVR | R-value | | -0.165 |
|  | P-value (two-tail) | | 0.017 |
| Max FDG SUVR | R-value | | -0.157 |
|  | P-value (two-tail) | | 0.024 |

Notes: Partial correlation analysis is used to explore the relationship between baseline NfL concentration and Aβ deposition. The coefficient of partial correlation, denoted as R-value, measures the strength and direction of a linear relationship between baseline NfL concentration and Aβ deposition, while controlling for the effect of age, sex and education years.

Abbreviations: FDG, 18-Fluorodeoxyglucose; SUVR, standardized uptake value ratio.

**Supplementary Table.4 Associations between NfL and brain volume**

| Regions |  | |
| --- | --- | --- |
| Right Amygdala | R-value | -0.279 |
|  | P-value (two-tail) | 0.001 |
| Right Hippocampus | R-value | -0.262 |
|  | P-value (two-tail) | 0.002 |
| Left Amygdala | R-value | -0.322 |
|  | P-value (two-tail) | 0.000 |
| Left Hippocampus | R-value | -0.271 |
|  | P-value (two-tail) | 0.001 |
| Left_Bankssts | R-value | -0.169 |
|  | P-value (two-tail) | 0.044 |
| Left_Entorhinal | R-value | -0.199 |
|  | P-value (two-tail) | 0.018 |
| Left_Inferior Parietal | R-value | -0.287 |
|  | P-value (two-tail) | 0.001 |
| Left_Inferior Temporal | R-value | -0.235 |
|  | P-value (two-tail) | 0.005 |
| Left_Middle Temporal | R-value | -0.299 |
|  | P-value (two-tail) | 0.000 |
| Left_Precuneus | R-value | -0.193 |
|  | P-value (two-tail) | 0.021 |
| Right_Inferior Temporal | R-value | -0.232 |
|  | P-value (two-tail) | 0.006 |
| Right_Middle Temporal | R-value | -0.221 |
|  | P-value (two-tail) | 0.008 |

Notes: Partial correlation analysis is used to explore the relationship between baseline NfL concentration and Aβ deposition. The coefficient of partial correlation, denoted as R-value, measures the strength and direction of a linear relationship between baseline NfL concentration and Aβ deposition, while controlling for the effect of age, sex and education years.

| **Supplementary Table.5 Associations between the rate of change in plasma NfL and brain volume.** | | |
| --- | --- | --- |
| Regions | P values | t values |
| **Cortical volume** | | |
| Left inferior parietal cortex | 0.043 | -2.04 |
| Left inferior temporal cortex | 0.047 | -2.00 |
| Left lateral occipital cortex | 0.047 | -2.00 |
| Left lateral orbitofrontal cortex | 0.033 | -2.15 |
| Left middle temporal cortex | 0.008 | -2.69 |
| Left para-hippocampal cortex | 0.020 | -2.35 |
| Left superior frontal cortex | 0.012 | -2.53 |
| Right frontal pole cortex | 0.027 | 2.23 |
| Right inferior parietal cortex | 0.003 | -3.06 |
| Right inferior temporal cortex | 0.010 | -2.60 |
| Right lateral occipital cortex | 0.031 | -2.18 |
| **Subcortical Volume** | | |
| Left hippocampus | 0.001 | -3.54 |
| Right hippocampus | 0.013 | -2.51 |
| Right accumbens area | 0.016 | 2.42 |
| **Hippocampal subfields volume** | | |
| Right CA2_3 | 0.036 | -2.11 |
| Right CA4_DG | 0.047 | -2.00 |
| Right Presubiculum | 0.035 | -2.12 |
| Right subiculum | 0.002 | -3.08 |

Notes: multivariate linear mixed effects model is used to explore the relationship between the change of plasma NfL concentrations and brain volume, while controlling for the effect of age, sex and education years.

Abbreviations: DG, Dentate Gyrus.

| **Supplementary Table.6 Associations between the rate of change in plasma NfL and DTI metrics** | | |
| --- | --- | --- |
| DTI metrics | P values | t values |
| **AD** | | |
| Left corticospinal tract | 0.002 | 3.23 |
| Left inferior cerebellar peduncle | 0.001 | 3.47 |
| Left superior cerebellar peduncle | 0.003 | 3.18 |
| Left superior fronto-occipital fasciculus | 0.002 | 3.22 |
| Right medial lemniscus | 0.021 | 2.39 |
| Right corticospinal tract | 0.000 | 3.94 |
| Right cerebral peduncle | 0.021 | 2.39 |
| **MD** | | |
| Left corticospinal tract | 0.000 | 3.95 |
| Left inferior cerebellar peduncle | 0.001 | 3.39 |
| Left medial lemniscus | 0.003 | 3.10 |
| Left cerebral peduncle | 0.001 | 3.70 |
| Left posterior thalamic radiation | 0.015 | 2.51 |
| Left superior fronto-occipital fasciculus | 0.001 | 3.55 |
| Right corticospinal tract | 0.000 | 4.07 |
| Right medial lemniscus | 0.002 | 3.32 |
| Right cerebral peduncle | 0.004 | 2.99 |
| Right cingulum | 0.038 | 2.13 |
| Bilateral full corpus callosum | 0.035 | 2.17 |
| **RD** | | |
| Left corticospinal tract | 0.000 | 4.08 |
| Left inferior cerebellar peduncle | 0.002 | 3.26 |
| Left medial lemniscus | 0.001 | 3.47 |
| Left cerebral peduncle | 0.001 | 3.71 |
| Left posterior thalamic radiation | 0.008 | 2.77 |
| Left superior fronto-occipital fasciculus | 0.001 | 3.67 |
| Left external capsule | 0.043 | 2.07 |
| Left splenium of corpus callosum | 0.036 | 2.16 |
| Right corticospinal tract | 0.000 | 4.03 |
| Right medial lemniscus | 0.000 | 3.84 |
| Right anterior limb of internal capsule | 0.024 | 2.32 |
| Right sagittal stratum | 0.043 | 2.07 |
| Bilateral splenium of the corpus callosum | 0.041 | 2.10 |
| Bilateral full corpus callosum | 0.016 | 2.49 |
| **FA** | | |
| Left corticospinal tract | 0.002 | -3.35 |
| Left anterior limb of internal capsule | 0.022 | 2.36 |
| Left posterior limb of internal capsule | 0.044 | 2.06 |
| Left posterior thalamic radiation | 0.002 | -3.22 |
| Left superior longitudinal fasciculus | 0.037 | -2.14 |
| Left splenium of corpus callosum | 0.010 | -2.68 |
| Bilateral splenium of the corpus callosum | 0.020 | -2.41 |

Notes: multivariate linear mixed effects model is used to explore the relationship between the change of plasma NfL concentrations and brain atrophy, while controlling for the effect of age, sex and education years.

Abbreviations: AD, axial diffusion; MD, mean diffusivity; RD, radial diffusion; FA, fractional anisotropy.
